# Suitability of a Fluorescence Method for Flavin Mononucleotide Determination in Modified HTK-Based Perfusion Solutions During Hypothermic Oxygenated Perfusion of Liver Grafts

**DOI:** 10.3390/mps9040122

**Published:** 2026-08-19

**Authors:** Yulia B. Basok, Eugenia G. Kuznetsova, Aleksandra D. Belova, Dmitriy A. Morozov, Mikhail A. Boldyrev, Artem R. Monakhov, Nikita V. Grudinin, Vladimir K. Bogdanov, Denis M. Bondarenko, Stepan I. Zubenko, Nidzhat M. Yusuf, Sergey V. Gautier

**Affiliations:** 1 Shumakov National Medical Research Center of Transplantology and Artificial Organs, 1 Shchukinskaja St., Moscow 123182, Russia; kuzeugenia@yandex.ru (E.G.K.); sashak1994@mail.ru (A.D.B.); morozov-d-a@mail.ru (D.A.M.); comex.ksb@gmail.com (M.A.B.); a.r.monakhov@gmail.com (A.R.M.); zbignev.religa@mail.ru (N.V.G.); bogdanovv@bk.ru (V.K.B.); bdm_2004@mail.ru (D.M.B.); petrasevivaninchuk@gmail.com (S.I.Z.); ynidzhat@gmail.com (N.M.Y.); priemtranspl@yandex.ru (S.V.G.); 2 The Sechenov University, 8-2 Trubetskaya St., Moscow 119991, Russia

**Keywords:** flavin mononucleotide, FMN, perfusion solution, hypothermic oxygenated machine perfusion, HOPE, liver transplantation

## Abstract

A comprehensive assessment of allograft quality is essential for predicting successful transplantation and graft survival. Elevated levels of flavin mononucleotide (FMN) in perfusate obtained during hypothermic oxygenated perfusion (HOPE) may serve as a promising predictor of early graft dysfunction and post-transplant complications. The aim of this study was to evaluate the feasibility of applying an FMN determination method to HTK-based perfusion solutions used during liver machine perfusion. The spectrofluorimetric method for FMN determination in modified HTK perfusion solution (modHTK) was evaluated for the following parameters: linearity, accuracy, repeatability, reproducibility, and stability. The study demonstrated the suitability of the quantitative FMN determination method for modHTK solution used during HOPE of liver grafts.

## 1. Introduction

Liver transplantation remains the only therapeutic option for end-stage liver disease. However, the shortage of suitable donor grafts and the growing number of patients on waiting lists remain key problems of modern transplantation, which can be solved by expanding the criteria for donor graft suitability [1,2].

The use of donor grafts after prolonged static cold preservation (more than 11–12 h), as well as grafts from extended-criteria donors (ECD), is associated with an increased risk of primary allograft dysfunction, in whose pathogenesis ischemia–reperfusion injury (IRI) plays a key role. IRI of a liver graft occurs to some degree in every liver transplantation and represents one of the principal factors impairing its early postoperative function. Therefore, improving early graft function and minimizing the consequences of preservation remain important objectives [3].

Machine perfusion of solid organs—normothermic machine perfusion (NMP), hypothermic oxygenated perfusion (HOPE; mono-HOPE or dual-HOPE (DHOPE))—is gaining increasing importance in transplantation not only because of its ability to reduce IRI, but also because it enables assessment of graft viability prior to transplantation [4,5,6].

The successful use of organ machine perfusion depends on, in part, the composition of the perfusate, whose primary purpose is to prevent cellular edema, protect the graft from IRI, and extend the allowable safe preservation time. The Belzer UW MPS (Machine Perfusion Solution), based on potassium lactobionate and hydroxyethyl starch and commonly known as the University of Wisconsin solution, is the “gold standard” and one of the most widely used solutions in liver machine perfusion in transplantation [7]. Custodiol HTK (histidine–tryptophan–ketoglutarate) has a lower viscosity compared with Belzer UW because it does not contain hydroxyethyl starch, which allows faster flushing and cooling of the donor organ.

A retrospective study showed that use of Custodiol HTK was associated with a lower incidence of biliary strictures after liver transplantation compared with Belzer UW [8]. The efficacy of using an HTK-based perfusate for D-HOPE and an erythrocyte suspension-based perfusate for NMP was previously demonstrated [9].

Comprehensive assessment of allograft quality is vital for predicting successful transplantation and long-term graft survival. However, to date there is no validated scoring system or biomarker that is widely used in clinical practice by transplant teams to guide decisions about graft suitability. Consequently, there is a need for reliable graft-quality assessment methods that provide rapid, real-time results [10].

Flavin mononucleotide (FMN), also known as riboflavin-5′-phosphate, is an autofluorescent coenzyme and the main form of riboflavin (vitamin B2) in cells and tissues, oxidizes NADH and provides electrons for ubiquinone reduction. It is a constituent of mitochondrial complex I, which plays a central role in the mitochondrial respiratory chain. During ischemia the noncovalent binding between reduced FMN and mitochondrial complex I weakens. Upon reperfusion, reverse electron transfer, driven by succinate, leads to FMN dissociation and generation of reactive oxygen species. Loss of FMN reduces complex I activity and thus causes mitochondrial dysfunction; FMN is released from damaged mitochondria [11,12,13,14,15,16].

In most eukaryotic cells mitochondria efficiently generate chemical energy (adenosine triphosphate) via oxidative phosphorylation. In liver ischemia/reperfusion, excessive production of reactive oxygen species and increased mitochondrial membrane permeability leading to mitochondrial dysfunction are principal causes of hepatocyte death—the main functional parenchymal cells of the liver—and of IRI [17].

Several research groups have demonstrated that FMN levels in perfusate samples collected during HOPE are promising predictors of graft survival and post-transplant complications [13,18,19]. Despite its potential, widespread implementation of quantitative FMN assessment in perfusate samples as a decision-making biomarker for organ transplantation has been delayed due to heterogeneous methodologies and non-standardized threshold values for FMN concentration. Moreover, commonly used methods such as enzyme-linked immunoassays and liquid chromatography-tandem mass spectrometry, which enable quantitative determination of biomarkers, are costly and time-consuming due to complex sample preparation, preventing real-time measurement of FMN levels [10]. Importantly, FMN measurement is not intended to diagnose hepatic steatosis, which is assessed by histological examination, but rather to provide additional information on mitochondrial injury and graft metabolic status during machine perfusion.

The aim of this study was to evaluate the feasibility of applying an FMN determination method to modified HTK-based perfusion solutions used during machine perfusion of the liver.

## 2. Materials and Methods

### 2.1. Stock and Working Solutions

A modified HTK solution (modHTK) was used as the perfusate. Composition of the modHTK: 25% albumin, 400 mL; N-acetylcysteine, 1500 mg; mannitol, 400 mL; sodium bicarbonate 4.8%, 200 mL; methylprednisolone, 1000 mg; maxictam, 1000 mg; vasastenon, 60 µg; nitroglycerin, 20 mg; insulin R, 30 IU.

A stock FMN solution with a concentration of 10 µg/mL was prepared by mixing FMN standard (riboflavin mononucleotide, injection solution 10 mg/mL; JSC “Pharmstandard-UfaVITA”, Ufa, Russia) with modHTK in a 1:1000 ratio. To prepare a working solution with FMN concentration of 1 µg/mL, the stock solution (10 µg/mL) was diluted 1:10 with modHTK perfusion solution.

### 2.2. Identification of FMN in Perfusion Solutions

UV–visible absorption spectra were recorded using a benchtop double-beam spectrophotometer Shimadzu UV-2600 (Shimadzu Corp., Kyoto, Japan) with the UVProbe software, version 2.42. Measurements were performed over the 200–500 nm spectral range using two quartz cuvettes with an optical path length of 10 × 10 mm and a volume of 3.5 mL.

Fluorescence spectroscopy was used to determine FMN concentration in HTK and modHTK perfusion solutions. Fluorescence signal intensity (in relative fluorescence units, RFU) was measured using a Tecan Spark 10M microplate reader with Spark Control Magellan V1.2.20 software (Tecan Group Ltd., Zürich, Switzerland) and 96-well black microplates (SPL Lifesciences Co., Ltd., Pocheon-si, Republic of Korea). Exactly 50 µL of sample and 150 µL of perfusion solution were added to each well (total volume per well 200 µL).

### 2.3. Testing of the Fluorescence Method for Determining FMN

The spectrofluorimetric method for FMN determination in modHTK was evaluated for the following parameters: linearity, accuracy, repeatability, reproducibility, and stability. The method was tested in accordance with the guidelines described in the International Council for Harmonisation guideline ICH Q2(R2) [20].

#### 2.3.1. Linearity

Linearity was defined as the ability of the method to produce results directly proportional to the FMN concentration in model samples over a specified range. A linear relationship between analyte concentration and response should be evaluated across the range of the analytical procedure to confirm the suitability of the procedure for the intended purpose [20].

For this purpose, seven solutions with the following FMN concentrations were tested: 0.0; 0.5; 0.25; 0.125; 0.0625; 0.03125; and 0.015625 µg/mL. Samples were prepared by serial 1:1 dilution of the working solution (FMN concentration 1 µg/mL) with modHTK perfusion solution. The fluorescence signal intensity of the blank perfusion solution was taken as the zero FMN concentration. The resulting data were subjected to statistical analysis and the correlation coefficient (R^2^) was calculated; it was required to be at least 0.98.

#### 2.3.2. Accuracy

Accuracy studies should be demonstrated as the closeness of agreement between the value found and the true value or an accepted reference value across the specified range of the analytical procedure. Accuracy should be demonstrated through comparison of the measured results with expected values. Accuracy should be demonstrated under regular test conditions of the analytical procedure [20].

Five model samples were tested with the following FMN concentrations: 0.20; 0.25; 0.30; 0.35; 0.40 µg/mL. Samples were prepared by diluting the FMN standard (10 mg/mL) with modHTK to the required concentrations. Fluorescence intensity was then measured, and FMN concentrations were determined using the previously constructed calibration curve. As the acceptance criterion for method accuracy, the standard deviation of the mean result for quantitative FMN determination, calculated using Formula (1), was required to be less than 2%.

S_x_ = (S × 100%)/(sqrt(n) × x), (1)

where S—standard deviation; n—number of measurements; x—mean value.

#### 2.3.3. Repeatability and Reproducibility

For the repeatability study, the same model solution (0.3 µg/mL FMN in modHTK) was analyzed five times by the same operator under identical conditions (equipment, reagents). Repeatability was evaluated by the mean result, whose deviation from the nominal concentration should not exceed ± 2%, and by the standard deviation, which should not exceed 2%.

Method reproducibility was evaluated by analyzing the same solution (0.3 µg/mL FMN in modHTK) by three different operators on different days. Reproducibility was assessed by the mean value, whose deviation from the nominal concentration should not exceed ±2%, and by the standard deviation, which should not exceed 3%.

#### 2.3.4. Stability

The stability of model samples (0.2–0.5 µg/mL FMN in modHTK solution) was studied during storage in a refrigerator (4 °C) for 6 days. The stability of actual FMN perfusion solutions obtained during HOPE was studied during storage in a frozen state (−18 °C) for 1, 9, 11, and 12 months. During storage of FMN solution samples, its concentration must not change by more than 2% from the initial concentration.

### 2.4. Human Sample Preparation

A standard set of consumables and cardiopulmonary bypass equipment routinely used in cardiac surgery was used for perfusion. The material and technical support included a cardiopulmonary bypass machine (Sorin Stockert S5, LivaNova PLC, London, UK), a temperature-regulation unit Stockert 3T (LivaNova PLC, London, UK), an Affinity NT oxygenator (Medtronic PLC, Minneapolis, MN, USA), and a temperature-control console with tubing set. Continuous recirculation of the perfusate was maintained at a flow rate of 1 L/min [9].

#### 2.4.1. Sample Collection

**Figure 1:**
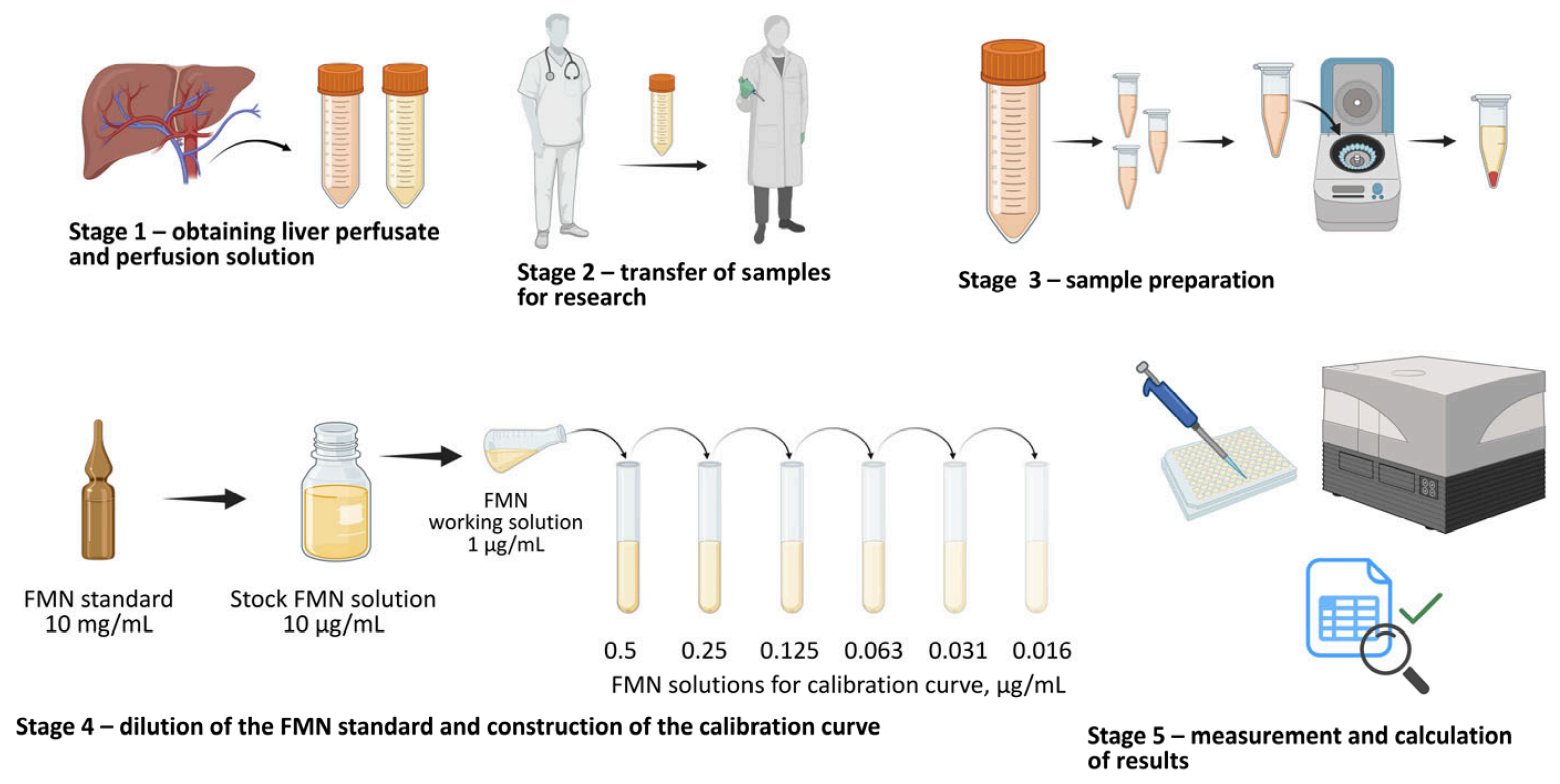
Flowchart of FMN fluorescence analysis. Created in BioRender. Belova, A. (2026).

Procedures related to sample preparation of perfusion solution, preparation of calibration standards, construction of the calibration curve, and quantitative determination of FMN in control and experimental samples are summarized in Figure 1.

Perfusate samples were collected during HOPE at 30 and 60 min after the start of perfusion according to the sampling scheme adopted at our center. Samples were transferred into 2 mL Eppendorf tubes (Wuxi NEST Biotechnology Co., Ltd., Wuxi, China) and centrifuged (centrifuge 5804R, Eppendorf SE, Hamburg, Germany) at 3000 rpm for 10 min to pellet cells, cellular debris, and insoluble particles that could interfere with FMN measurement. The supernatant was then carefully removed without disturbing the pellet.

For clinical samples in which the expected FMN concentration exceeded the upper calibration point (0.5 µg/mL), the sample was pre-diluted with the original modHTK solution prior to measurement, and the final concentration was then calculated taking the dilution factor into account.

The work was approved by the Local Ethics Committee at the Shumakov National Medical Research Center of Transplantology and Artificial Organs, Moscow, Russia (16 January 2025, Protocol No. 160125-1-1э).

#### 2.4.2. FMN Calibration Curve

Calibration standards were prepared by serial 1:1 dilutions of the working solution (FMN concentration 1 µg/mL) with modHTK perfusion solution. The calibration curve comprised the following FMN concentrations: 0.5, 0.25, 0.125, 0.0625, 0.03125, and 0.015625 µg/mL. The fluorescent intensity of the primary filling solution was taken as the zero FMN concentration.

The calibration curve was constructed from seven points, each obtained as the mean value of seven fluorescence intensity measurements of the calibration samples. Mean values and standard deviations were calculated. The results were plotted against the corresponding FMN concentrations and analyzed by simple linear regression. The linear regression equation and the mean fluorescence intensity of each experimental sample were used to calculate FMN concentrations in the experimental samples.

### 2.5. Statistical Analysis

Data were analyzed with SPSS26.0 statistical software. The distribution of variables was tested with the Shapiro–Wilk procedure. The results were compared using the Mann–Whitney unpaired U-test and Kruskal–Wallis test, where *p* < 0.05 was considered statistically significant.

## 3. Results 

FMN is of particular interest as a potential biomarker because, unlike conventional markers of cellular injury, it may reflect mitochondrial dysfunction rather than established cell damage. This is especially relevant because mitochondrial dysfunction is one of the key mechanisms underlying IRI in the graft.

### 3.1. Absorption Spectra of Perfusion Solutions and FMN

**Figure 2:**
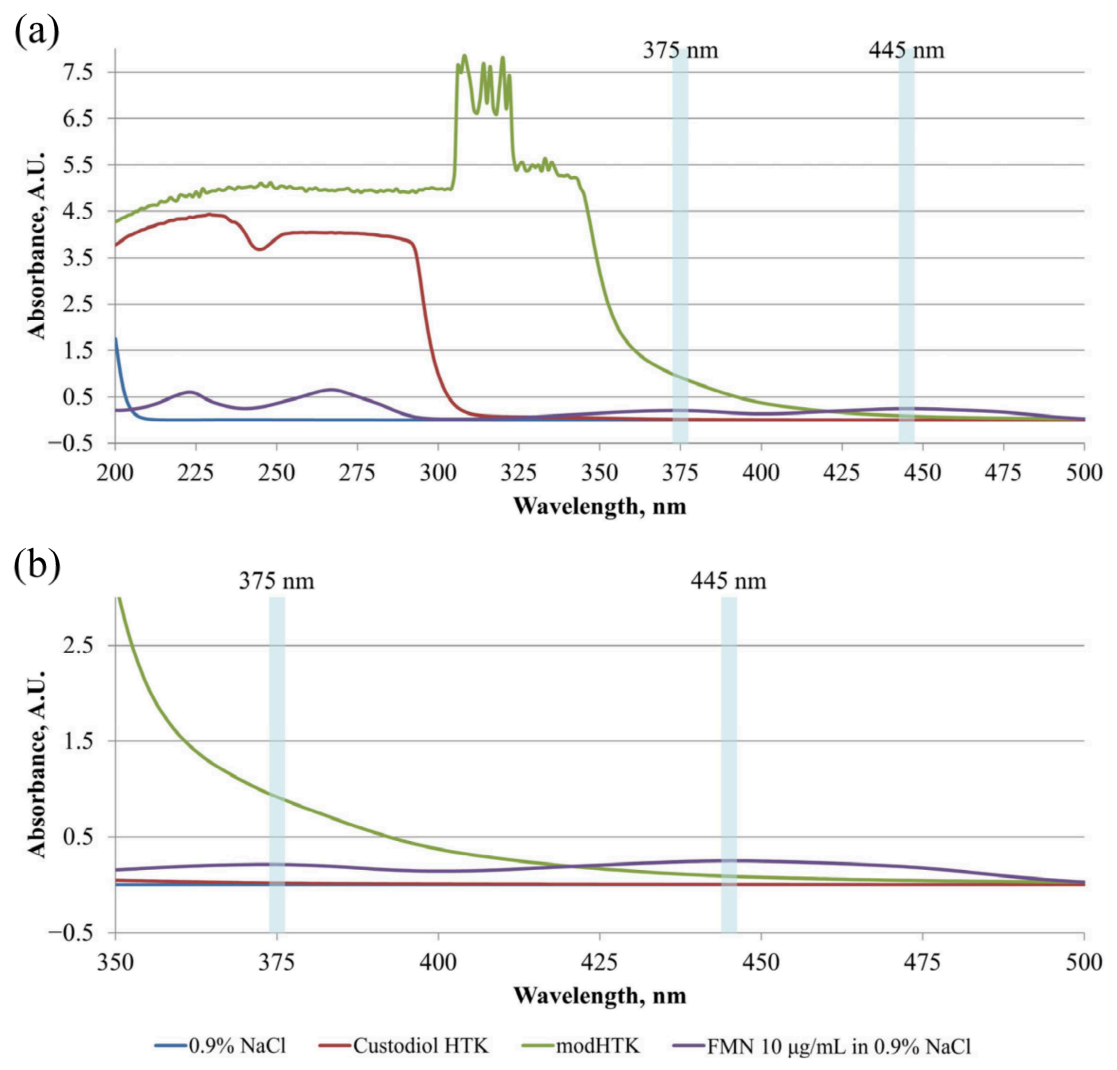
Absorption spectra of 0.9% NaCl, Custodiol HTK and modHTK solutions compared with FMN at a concentration of 10 μg/mL in 0.9% NaCl in the near-UV and visible blue range. Wavelength ranges: 200–500 nm (**a**) and 350–500 nm (**b**).

The absorption spectrum of FMN lies in the near-ultraviolet and visible regions and spans wavelengths from 320 to 500 nm and is characterized by two main absorption bands in acidic and neutral media at 375 nm and 445 nm [21]. For quantitative assessment of FMN concentration during machine organ perfusion it is necessary to distinguish potential factors that affect absorbance and fluorescence, which may be related both to the analyte itself and to components of the perfusion solutions. Therefore, we measured the absorption spectra of 0.9% NaCl, the standard Custodiol HTK, and modHTK perfusion solution over the wavelength range 200–500 nm (Figure 2).

The Custodiol HTK and modHTK perfusion solutions strongly absorb light in the ultraviolet range (up to 400 nm), especially at wavelengths below 300 nm for Custodiol HTK and below 360 nm for modHTK. The observed differences in absorbance can be attributed to composition- and concentration-related factors. The composition of modHTK is described in Section 2.1.

**Figure 3:**
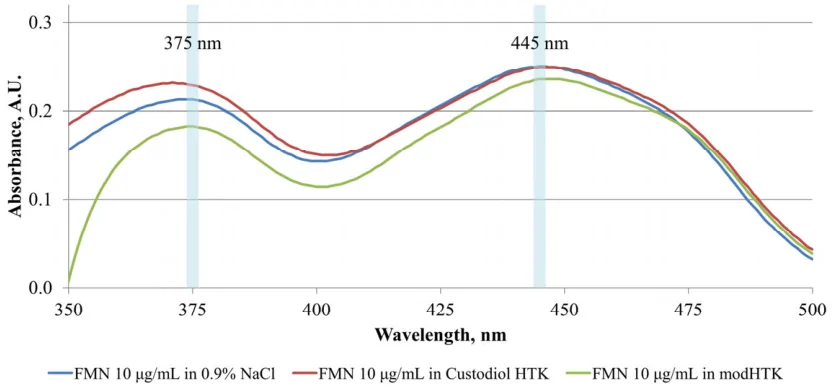
Absorption spectra of FMN in the near-UV and visible blue range.

When FMN was diluted in different perfusion solutions, the shape of the absorption spectra was identical to that in physiological saline (Figure 3). The absorbance values of FMN in 0.9% NaCl corresponded to those measured in Custodiol HTK and modHTK perfusion solutions.

The absence of spectral deviations such as peak shifts, band broadening, or loss of intensity suggests that there is no appreciable interaction between FMN and components of the perfusion solutions that would interfere with FMN quantification.

**Figure 4:**
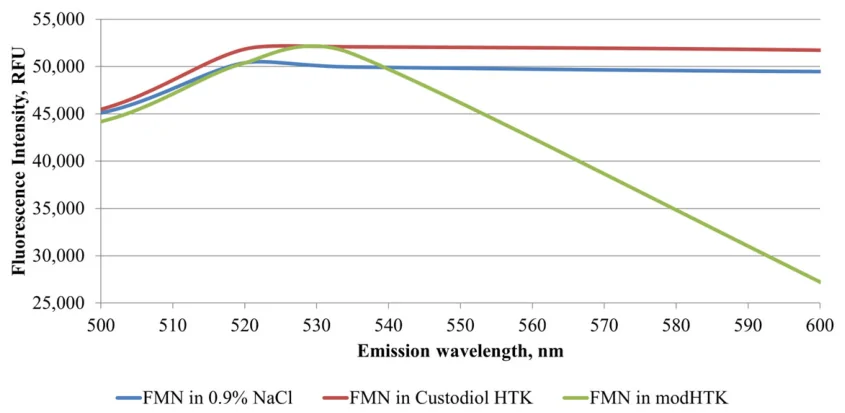
Fluorescence of FMN (concentration 0.25 μg/mL) in 0.9% NaCl, Custodiol HTK and modHTK solutions.

The fluorescence intensity of FMN at a concentration of 0.25 µg/mL when added to 0.9% NaCl, Custodiol HTK, and modHTK is shown in Figure 4.

The shape of the FMN fluorescence spectra remains unchanged when FMN is added to 0.9% NaCl and to Custodiol HTK perfusion solution; however, when added to modHTK perfusion solution the fluorescence signal intensity decreases. This decrease may be related to differences in solution composition.

**Figure 5:**
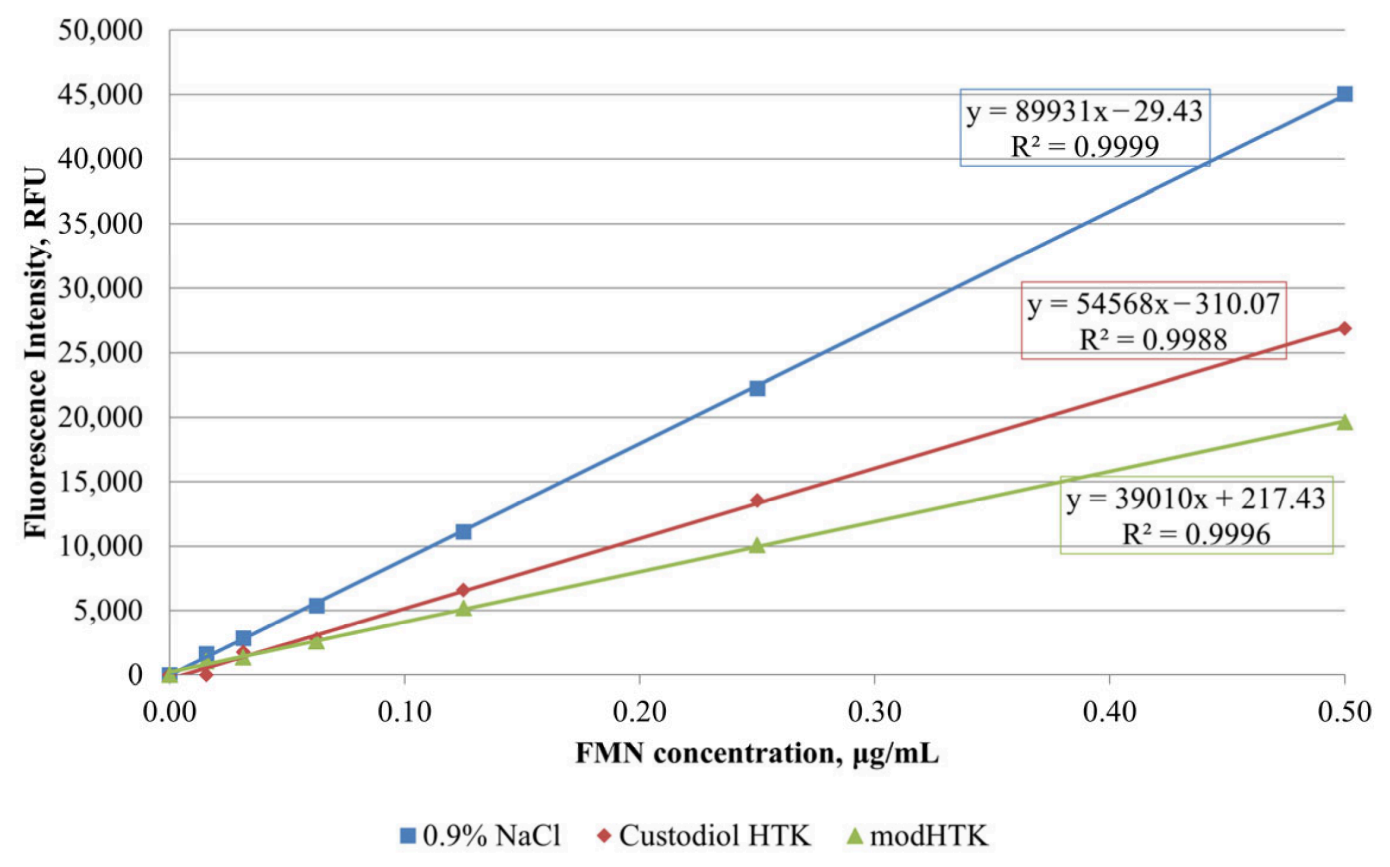
FMN fluorescence in NaCl 0.9%, Custodiol HTK and modHTK solutions.

FMN fluorescence in 0.9% NaCl, Custodiol HTK, and modHTK solutions at an excitation/emission wavelength of 485/520 nm is shown in Figure 5.

The data in Figure 5 clearly show that 0.9% NaCl and the Custodiol HTK and modHTK perfusion solutions have different spectral characteristics, resulting in different calibration curves. Therefore, when constructing a calibration curve for FMN quantification in perfusates obtained after perfusion, the original perfusion solution modHTK—prepared immediately before the start of perfusion—should be used.

### 3.2. Accuracy

**Figure 6:**
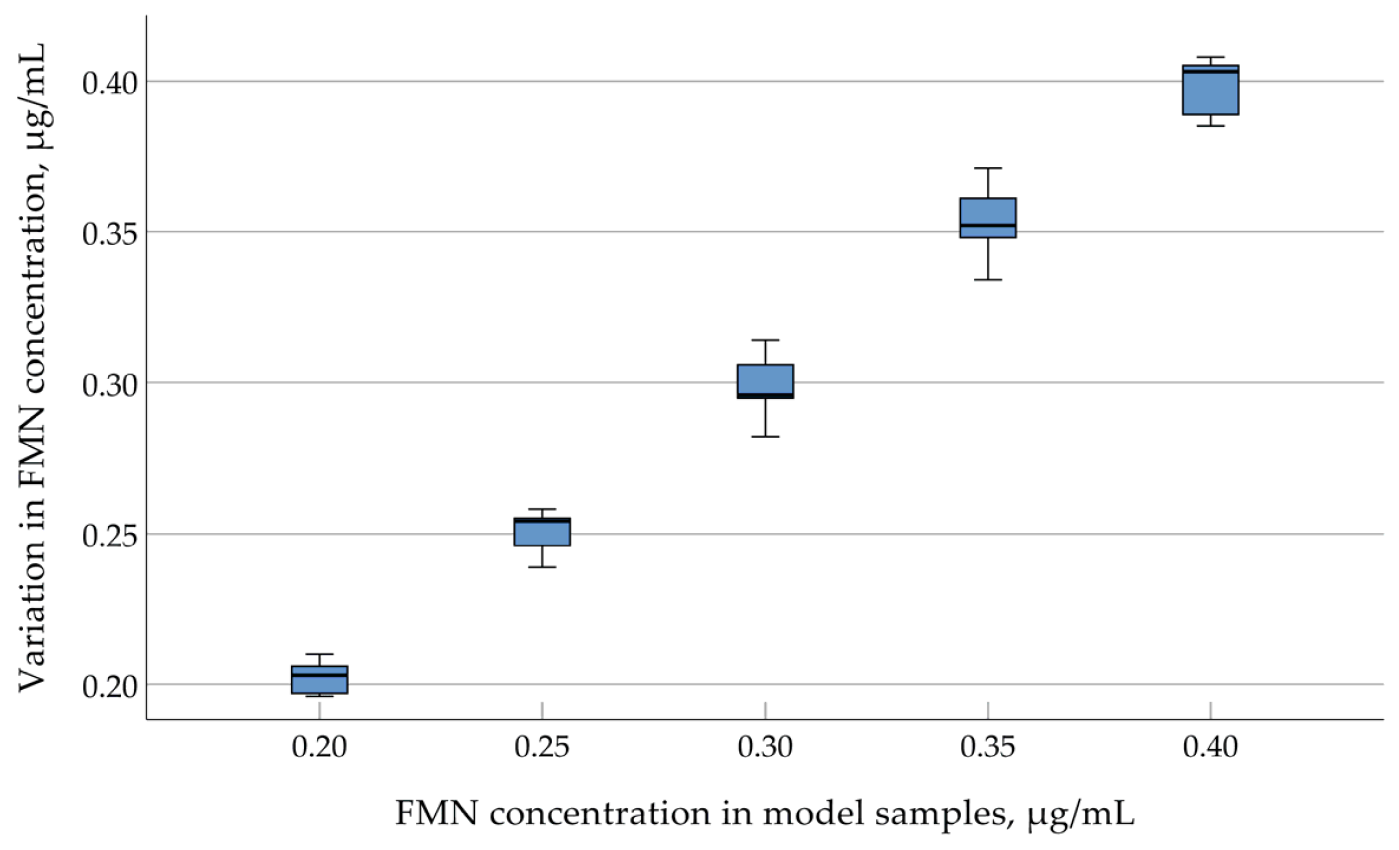
Assessment of the “Accuracy” parameter for the FMN assay.

Table 1 and Figure 6 present the results of quantitative determination of FMN in solutions of various concentrations to confirm the “Accuracy” test.

Table 1 shows that the standard deviation of the mean value was less than 2% for each solution with a known FMN concentration, which meets the test criteria.

Figure 6 illustrates the distribution of measured concentrations for the five solutions.

### 3.3. Linearity

**Figure 7:**
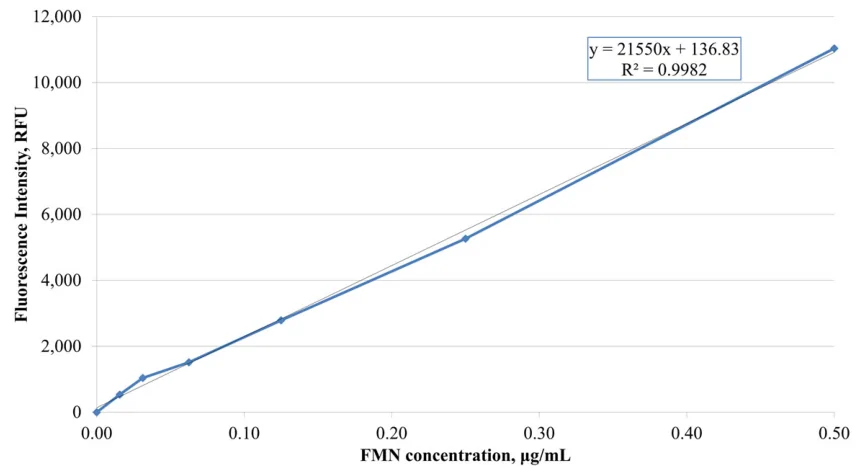
Assessment of the “Linearity” parameter for the FMN assay.

Linearity was evaluated from the calibration curve constructed from seven points, each obtained as the mean value of seven fluorescence intensity measurements of calibration samples with concentrations from 0 µg/mL to 0.5 µg/mL. Mean values, and standard deviations were calculated (Table 2). Results were plotted and fitted to the representative concentration using simple linear regression (Figure 7). The linear regression equation and the mean fluorescence intensity of each experimental sample were used to calculate FMN concentrations in the test samples.

As shown in Table 2, a good linear relationship was observed across the tested concentration range, with regression coefficients (R^2^) equal to 0.99.

### 3.4. Repeatability and Reproducibility

**Figure 8:**
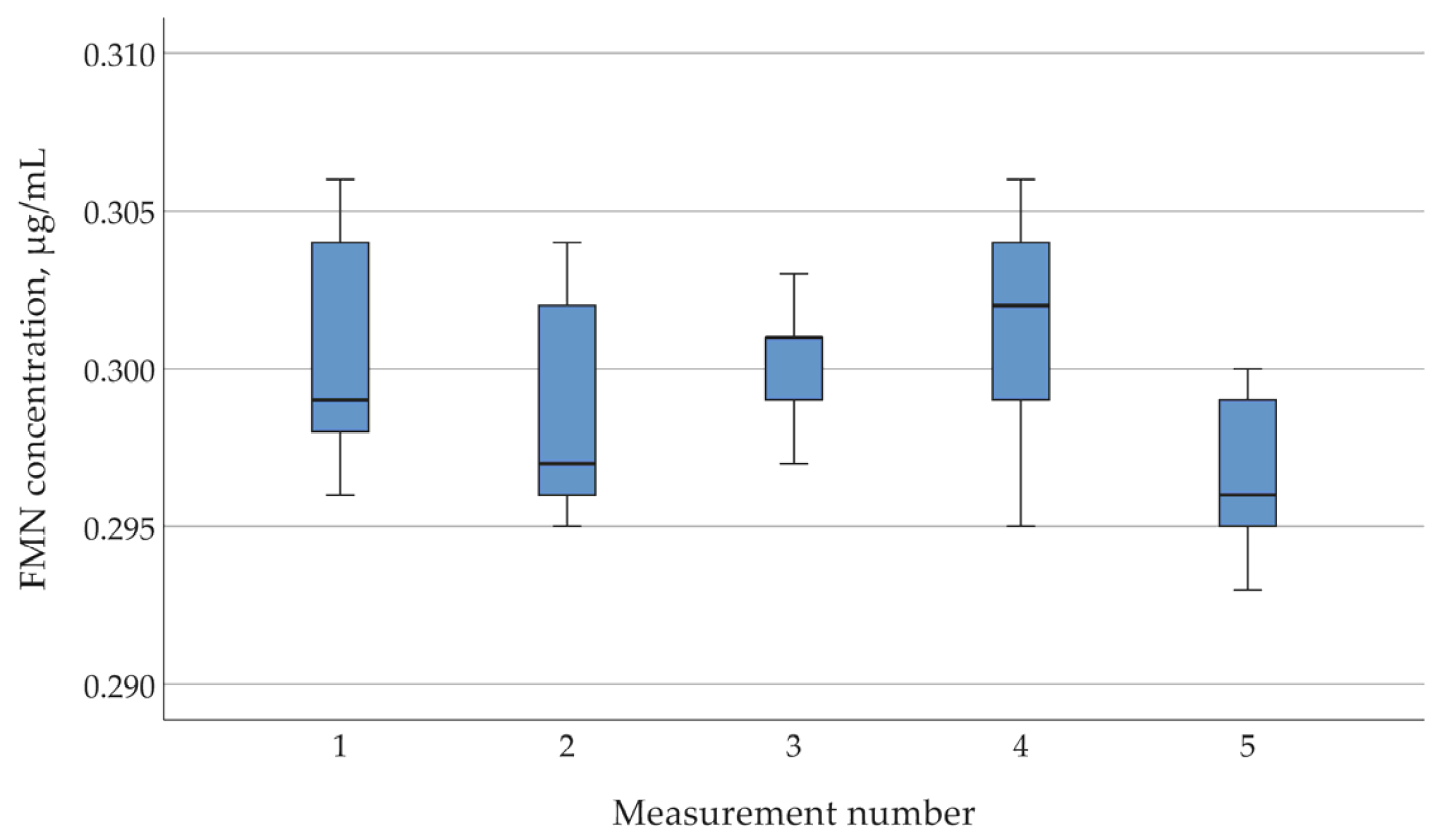
Assessment of the “Repeatability” parameter for the FMN assay.

Results of the repeatability test are presented in Table 3 and Figure 8. For each measurement sequence, the operator constructed a new calibration curve. The concentration for each of five replicate fluorescence measurements was determined using the equation of the calibration function.

As shown in Table 3, the mean results of all five measurements ranged from 0.296 to 0.301 µg/mL, which does not exceed 2% of the nominal concentration of 0.3 µg/mL, and the standard deviation did not exceed 2%. These data meet the criteria for the “Repeatability” test.

**Figure 9:**
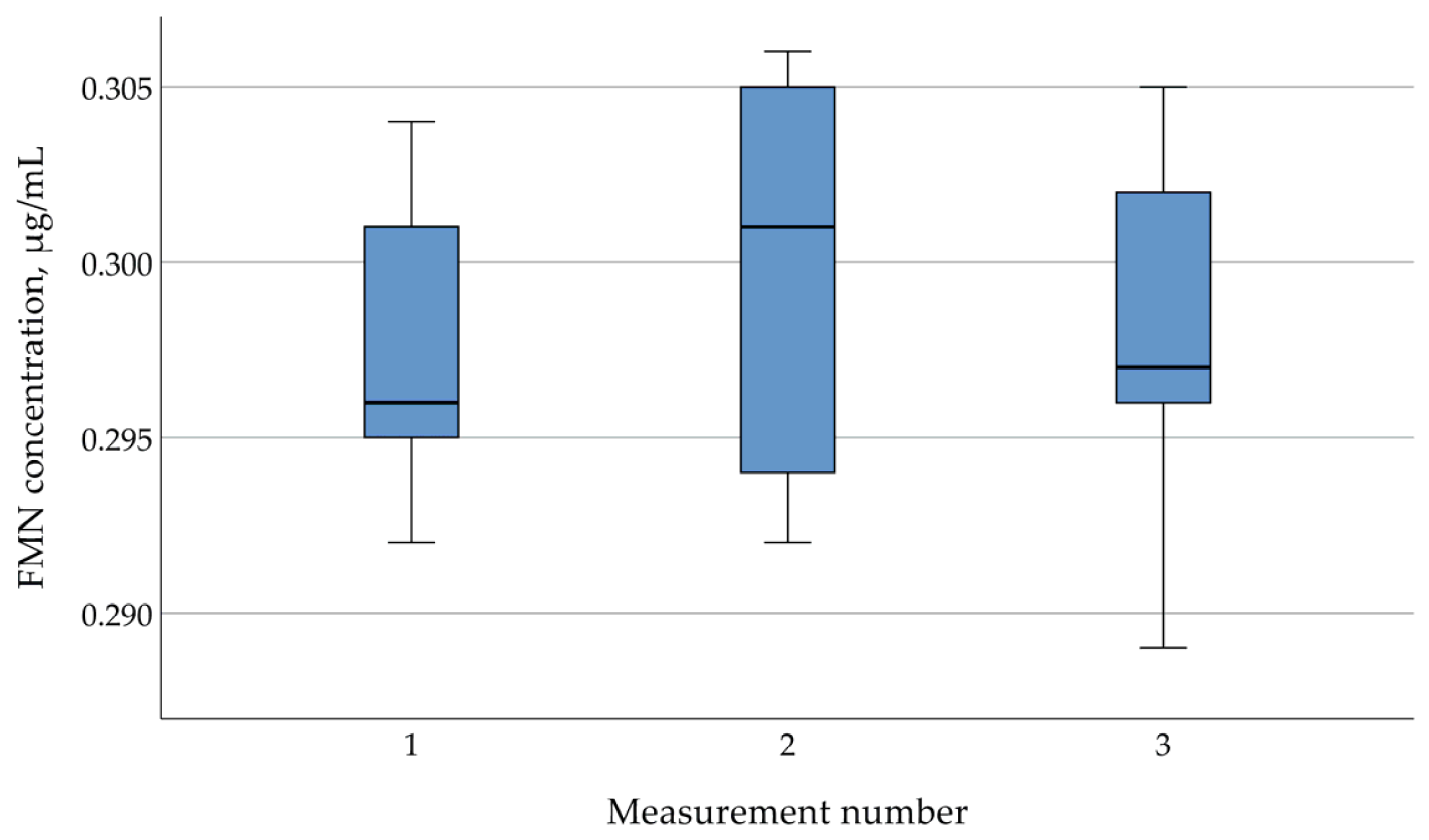
Assessment of the “Reproducibility” parameter for the FMN assay.

Results of the reproducibility test are presented in Table 4 and Figure 9. Measurements were performed by three operators, each of whom constructed a calibration curve of fluorescence signal versus FMN concentration.

As shown in Table 4, the mean results for two operators were 0.298 µg/mL and for one operator 0.299 µg/mL, which does not exceed 2% of the nominal concentration of 0.3 µg/mL, and the standard deviation did not exceed 3%. These data meet the criteria for the “Reproducibility” test.

### 3.5. Stability

Table 5 presents the results of quantitative determination of FMN concentration in modHTK perfusion solution of known concentration after storage for 6 days at 4 °C.

These data indicate that FMN concentration in the perfusion solution changed within 0.57% to 2% when samples were stored refrigerated at 4 °C for 6 days, which indicates that storage under these conditions is acceptable. 

The results of quantitative determination of FMN concentrations in actual perfusion solutions collected at 30 and 60 min of machine perfusion and stored frozen at −18 °C for 1, 9, 11, and 12 months are presented in Table 6.

As shown in Table 6 storage of samples at −18 °C for up to 1 month appears acceptable; whereas, long-term storage under these conditions is not recommended.

### 3.6. Human Samples

FMN measurement may be clinically relevant as an adjunctive marker of mitochondrial injury during HOPE in marginal grafts, including steatotic livers. In this context, FMN is not intended to diagnose steatosis itself, which is assessed histologically, but to complement the overall assessment of graft viability during perfusion.

We analyzed perfusate samples from six HOPE procedures collected at 30 and 60 min. Table 7 lists the parameters measured in the perfusate during the HOPE procedure.

As shown in Table 7, in the present small clinical series, higher FMN concentrations were observed in the two grafts with severe macrosteatosis; whereas, lower FMN values were found in grafts with absent or mild macrosteatosis. Given the small sample size, these findings should be interpreted cautiously and should not be understood as evidence that FMN can replace histological assessment of steatosis. Note that macrovesicular steatosis ≥ 50% in a donor liver is considered a predictor of graft failure; therefore, such organs are often classified as extended-criteria or marginal grafts [22]. In these perfusions, AST and ALT levels in perfusate were markedly higher than those observed in grafts No. 3–6. Notably, in cases No. 1 and No. 2 the liver grafts did not meet the viability criteria adopted at our center and therefore were not transplanted to the recipients [4]. It should be noted, in our study, AST and ALT levels in the perfusate were considered primarily as integrated markers of injury detected during perfusion, rather than as definitive evidence that this injury occurred specifically during HOPE.

Previous studies by Muller et al. and Ravaioli et al. suggested that FMN measured during HOPE may be associated with post-transplant outcomes [13,23]. In the present study, however, clinical observations were limited and did not include outcome-based validation. Therefore, our findings should be interpreted as preliminary and supportive of the analytical feasibility of FMN measurement in modHTK perfusate rather than as evidence of clinical prognostic utility”.

Thus, FMN measurement in perfusate collected during HOPE of liver allografts appears to have potential prognostic value for transplantation outcomes. However, this observation requires validation in a larger sample set and further research.

## 4. Discussion

FMN is of particular interest as a biomarker because, unlike conventional markers of graft injury that mainly reflect cellular disruption, it may more specifically indicate mitochondrial dysfunction. This distinction is highly relevant in the setting of IRI, where mitochondrial impairment is one of the central mechanisms of graft damage. Accordingly, FMN has emerged as a promising functional biomarker for assessing graft quality during machine perfusion and for supporting clinical decision-making in liver transplantation.

This study demonstrates that spectrofluorimetric quantification of FMN is feasible in modHTK, a modified HTK-based perfusion solution used during HOPE of liver grafts. The analytical validation showed that the method performs well in the concentration range relevant for perfusate analysis, with good linearity, acceptable accuracy, and satisfactory repeatability and reproducibility. 

Importantly, our findings also show that the fluorescence response of FMN is influenced by the composition of the perfusion matrix, underscoring the need for matrix-matched calibration when FMN is quantified in real HOPE samples. This point is critical for the clinical interpretation of FMN values. In practice, fluorescence intensity cannot be directly converted into concentration unless the calibration matrix closely matches the sample matrix. Without such matching, fluorescence readouts may be misleading and thresholds derived in one perfusion solution may not be transferable to another. This limitation is especially important in the setting of HOPE, where perfusion protocols and preservation solutions differ across centers. Our findings thus provide a methodological basis for standardizing FMN measurement in modHTK and emphasize that the perfusion solution itself must be accounted for when establishing quantitative assays.

The analytical validation confirmed that the assay is suitable for quantitative use. The calibration curve showed excellent linearity over the tested range from 0 to 0.5 µg/mL, with a coefficient of determination of 0.99. This range is clinically relevant because FMN levels observed during HOPE are expected to fall within low micromolar concentrations, and reliable detection at the lower end of the curve is therefore essential. Accuracy was also satisfactory, with deviations remaining within the predefined acceptance criteria and standard deviations below 2% for the tested concentrations. In addition, both repeatability and reproducibility were within acceptable limits, demonstrating that the method is robust not only within a single analytical run but also across different operators. Together, these results indicate that the assay can be implemented reliably in a translational transplant setting.

The clinical samples analyzed in this study provide preliminary evidence that FMN measurement in modHTK may reflect graft quality during HOPE. Higher FMN concentrations were observed in liver grafts with severe macrosteatosis; whereas, lower concentrations were detected in grafts with limited steatosis. This observation is biologically plausible, since steatotic livers are more vulnerable to hypoxic and reperfusion-related mitochondrial damage. In our cohort, the grafts with the highest FMN values also showed markedly elevated AST and ALT levels in the perfusate and did not meet the viability criteria used at our center. Although the number of cases was small, these findings support the concept that FMN release during perfusion may serve as a marker of mitochondrial injury and may contribute to viability assessment. A key point is that FMN should not be interpreted as a diagnostic marker of hepatic steatosis. Steatosis is more appropriately assessed by histological methods, which remain the reference methods for its grading. In our study, the apparent association between higher FMN concentrations and severe macrosteatosis was an exploratory observation in a very small number of grafts. This observation may reflect increased susceptibility of steatotic grafts to mitochondrial dysfunction and ischemia–reperfusion-related injury, but it does not demonstrate diagnostic accuracy for steatosis and does not substitute for post-transplant outcome analysis.

An important clinical consideration is that grafts with moderate macrosteatosis represent one of the most challenging categories in transplantation decision-making. While severely steatotic grafts are often regarded as high-risk, and grafts without steatosis are generally easier to evaluate, livers with moderate macrosteatosis may fall into a clinically uncertain zone in which additional viability markers may be particularly valuable. However, in the present study, the clinical series was too small and included too few grafts with moderate macrosteatosis to allow a meaningful subgroup analysis. Moreover, post-transplant outcome analysis was not performed. Therefore, our observations should be regarded as preliminary, and future studies should, in particular, include a larger number of grafts with moderate macrosteatosis and correlate perfusate FMN levels with transplantation outcomes.

Our results are consistent with the growing body of literature supporting FMN as a biomarker during machine perfusion. FMN released into the perfusate has been associated with graft injury and post-transplant outcomes in previous studies, including work by Müller et al., who reported that elevated FMN levels during HOPE were linked to worse clinical outcomes and graft loss, and by Ravaioli et al., who suggested that FMN measurement can provide useful prognostic information in the transplantation setting [13,23]. In addition, recent reports have strengthened the case for FMN as a real-time indicator of mitochondrial dysfunction during organ perfusion [14,15,16]. However, a limitation of some earlier studies is that FMN was reported as fluorescence signal rather than as an absolute concentration [13,24,25]. Since fluorescence readouts depend on the analytical system, matrix composition, and optical configuration, such values are not directly comparable across studies or platforms. By contrast, the present work establishes a quantitative framework for FMN analysis in modHTK and may help improve inter-study comparability.

Several limitations of the present study should be acknowledged. First, the number of human HOPE samples was limited, and the observed association between FMN concentrations and graft steatosis should therefore be considered exploring. Second, although the analytical performance of the assay was strong, clinically meaningful cut-off values for FMN in modHTK remain to be defined in larger prospective cohorts. 

Despite these limitations, the present study has several practical implications. The assay is relatively simple, rapid, and inexpensive, making it well suited for potential implementation in clinical workflows where timely decision-making is required. This is especially relevant in liver transplantation programs that rely on machine perfusion to assess marginal or extended-criteria donor grafts. A standardized FMN assay could help provide a more objective assessment of mitochondrial injury during HOPE and may contribute to improved graft selection and safer transplantation decisions.

In summary, this study shows that FMN can be measured quantitatively in modHTK perfusate with good analytical reliability. Further multicenter studies with larger cohorts are now needed to define clinically meaningful thresholds and to establish how FMN measurement can best be integrated into graft viability assessment during HOPE.

## 5. Conclusions

In the current study, we demonstrated the analytical feasibility of a spectrofluorimetric method for determining FMN concentration in modHTK perfusion solution used during HOPE of liver grafts. The clinical significance of perfusate FMN, including its relationship to graft viability and post-transplant outcomes, requires further evaluation in larger outcome-based studies.

## Figures and Tables

**Table 1:** Results of quantitative determination of FMN in solutions. Assessment of method accuracy.

№	FMN Concentration (μg/mL) in Model Samples				
	0.20	0.25	0.30	0.35	0.40
**1**	0.210	0.239	0.282	0.361	0.389
**2**	0.206	0.255	0.314	0.371	0.405
**3**	0.197	0.258	0.295	0.334	0.385
**4**	0.203	0.246	0.296	0.348	0.403
**5**	0.196	0.254	0.306	0.352	0.408
**x (mean value)**	0.202	0.250	0.299	0.353	0.398
**S (standard deviation)**	0.006	0.008	0.012	0.014	0.010
**S_x_ (standard deviation of the mean value)**	1.34%	1.43%	1.79%	1.77%	1.12%

**Table 2:** Results of quantitative determination of FMN in solutions. Assessment of method linearity.

№	FMN Concentration (μg/mL) in Model Samples/Fluorescence Intensity (RFU)					
	0.015625	0.03125	0.0625	0.125	0.250	0.500
**1**	39,001	39,390	40,705	41,416	43,917	49,530
**2**	38,956	39,541	39,859	41,357	44,456	50,011
**3**	39,016	39,505	40,026	41,613	43,631	49,063
**4**	39,006	39,751	40,030	41,051	43,962	49,849
**5**	38,892	39,541	39,645	40,854	42,891	49,064
**6**	39,056	39,213	39,537	41,145	43,432	49,399
**x (mean value)**	38,988	39,490	39,967	41,239	43,715	49,486
x_0_ (mean value minus background *)	536	1038	1515	2787	5263	11,034
**R^2^ (correlation coefficient)**	0.998					

* The average result minus the background—the fluorescence intensity value of a solution with a FMN concentration of 0 μg/mL.

**Table 3:** Results of quantitative determination of FMN in solutions. Assessment of method repeatability.

Test Sequence	1	2	3	4	5
**FMN concentration in the model sample, μg/mL**	**0.300**	**0.300**	**0.300**	**0.300**	**0.300**
**The obtained values of FMN concentration in solution, μg/mL**	0.298	0.296	0.303	0.306	0.296
	0.304	0.295	0.301	0.299	0.293
	0.299	0.304	0.299	0.295	0.295
	0.296	0.302	0.301	0.302	0.300
	0.306	0.297	0.297	0.304	0.299
**x (mean value)**	0.301	0.299	0.300	0.301	0.297
**S (standard deviation)**	0.004 (2%)	0.004 (2%)	0.002 (1%)	0.004 (2%)	0.003 (1.5%)

**Table 4:** Results of quantitative determination of FMN in solutions. Assessment of method reproducibility.

	Test Sequence		
	1st Operator	2nd Operator	3rd Operator
**FMN concentration in the model sample, μg/mL**	**0.300**	**0.300**	**0.300**
**The obtained values of FMN concentration in solution, μg/mL**	0.292	0.292	0.305
	0.304	0.306	0.289
	0.295	0.294	0.302
	0.296	0.301	0.296
	0.301	0.305	0.297
**x (mean value)**	0.298	0.299	0.298
**S (standard deviation)**	0.004 (2%)	0.006 (3%)	0.006 (3%)

**Table 5:** Results of quantitative determination of FMN in modHTK solution, storage at 4 °C for 6 days. Assessment of sample stability.

FMN Concentration in the Model Sample, μg/mL	0.200	0.250	0.300	0.350	0.400
**The obtained values of FMN concentration in solution, μg/mL**	0.203 ± 0.002	0.255 ± 0.001	0.304 ± 0.006	0.348 ± 0.005	0.407 ± 0.002
**Difference from known concentration, %**	1.50	2.00	1.30	0.57	1.80

**Table 6:** Results of quantitative determination of FMN in modHTK solution, storage at −18 °C. Assessment of sample stability.

Storage Time	1 Month		9 Months		11 Months		12 Months	
Perfusion time	30 min	60 min	30 min	60 min	30 min	60 min	30 min	60 min
**Initial concentration of FMN, μg/mL**	0.197 ± 0.041	0.251 ± 0.032	0.190 ± 0.020	0.230 ± 0.010	0.120 ± 0.010	0.250 ± 0.010	0.314 ± 0.021	1.173 ± 0.053
**FMN concentration after storage, μg/mL**	0.222 ± 0.014	0.249 ± 0.018	0.200 ± 0.021	0.300 ± 0.021	0.128 ± 0.014	0.404 ± 0.015	0.327 ± 0.044	0.793 ± 0.165

**Table 7:** Quantification of perfusate parameters during HOPE.

Parameters/Samples	1	2	3	4	5	6
**FMN in perfusate 30 min**	0.31	1.11	below the calibrated range	0.11	0.11	0.03
**FMN in perfusate 60 min**	1.17	1.12	below the calibrated range	0.15	0.10	0.05
**AST in perfusate, U/L**	4620	6101	150.8	178.3	218.8	199.6
**ALT in perfusate, U/L**	1926	1859	82.1	56.3	236.8	219
**Lactate in perfusate, mmol/L**	4.2	3.9	2.3	1.7	1.9	2.3
**Glucose in perfusate, mmol/L**	16.3	3.5	9.2	5.3	4.6	11.7
**Macrosteatosis, %**	95	80	30	0	0	0
**HOPE time, min**	129	111	60	170	115	216

## Data Availability

The original contributions presented in the study are included in the article, further inquiries can be directed to the corresponding author.

## Abbreviations

The following abbreviations are used in this manuscript:

ECD	Extended-criteria donors
IRI	Ischemia–reperfusion injury
NMP	Normothermic machine perfusion
HOPE	Hypothermic oxygenated perfusion
DHOPE	Dual hypothermic oxygenated perfusion
HTK	Histidine–tryptophan–ketoglutarate
FMN	Flavin mononucleotide
modHTK	Modified HTK solution
RFU	Relative fluorescence units
